# Supplementary material for: Phylogenomic analysis and Mycobacterium tuberculosis antibiotic resistance prediction by whole-genome sequencing from clinical isolates of Caldas, Colombia
Source: PLoS One. 2021 Oct 7;16(10):e0258402. doi: 10.1371/journal.pone.0258402 (PMC8496870; doi:10.1371/journal.pone.0258402)
Supplement: Attachment — Submitted filename: PONE-D-21-17481_Comments.doc [file pone.0258402.s002.doc]

**Phylogenomic analysis and *Mycobacterium tuberculosis* antibiotic resistance prediction**

**by whole-genome sequencing from clinical isolates of Caldas, Colombia**

The authors have carried out work entitled “Phylogenomic analysis and *Mycobacterium tuberculosis* antibiotic resistance prediction by whole-genome sequencing from clinical isolates of Caldas, Colombia.” The study has explored the variants present in MTB clinical samples from

Chinchiná, Caldas, a small town in Colombia with a high incidence of TB.

However, there is some gap in the experimental work to reach some significant outcome which authors need to address.

**Major comments:**

1. For the prediction of drug resistance causing variants against bedaquiline and delamanid drugs which are recently FDA approved drugs for the treatment of TB, the authors can consider recently published articles related to these drugs. They can report whether variants related to these drugs are present in the analyzed clinical samples.
2. The authors have reported that there sampes of HIV+TB (Co-morbidity) and TB patients. However, they have analyzed the samples in one group only. It will add significanse value to the study if they compare the variants of TB patients with patients having comorbdity condition.
3. Authors are giving synonymous variants. They should explain why and how does it affect the drug resistance development on MTB?
4. Authors have mentioned, “Likewise, for further analysis it would be considered to carry out protein modeling to determine if changes in the mutated amino acids could generate resistance.” However, the effect of variants on protein function can be analysed by SIFT (whether an amino acid substitution affects protein function ), PROVEAN (an amino acid substitution or indel has an impact on the biological function of a protein) or PolyPhen. Authors can add this step to their bioinformatic analysis.

**Minor comments:**

1. **Abstract**
1. “All samples were proven sensitive to antituberculosis drugs by phenotype and genomic analysis, although unreported mutations were identified.” Authors have mentioned phenotype, however they have not carried out Drug susceptibility testing, therefore this sentence should be modified, Line nos. 36-37; Page No. 2.

2. **Introduction**
1. Should be read as, “1.7 million” for “1,7 million”, Line no. 47; Page No. 3.
2. Should be read as, “100,000 habitants at” for “100.000 habitants at” Line no. 52; Page No. 3.
3. “Also, outbreaks can be identified with higher resolution, allowing optimized control measures not only by revealing the genetic makeup and evolution of strains, but by showing epidemiological evidence of contagion from patients unwilling or unable to give information for the study of the case, as its routinely the case for high-risk social groups like homeless, immigrants, and itinerant agricultural workers.” Sentence is too long. Rewrite it. Line no. 66; Page No. 3: Line nos. 67-70; Page No. 4.
2. **Materials and methods**
   2.1. **DNA extraction**
1. Should be read as, “visualization in” for “visualization by”, Line no. 96; Page No. 5.

**2.4.3 Variant calling and prediction of drug resistance.**

**1.** The authors have repeated this section from “The readings were reviewed [4], validated (FastQVAlidator v1.0.5 2017) and trimmed (Trimmomatic v0.36) and the processed reads were mapped using BWA v0.7.17 [19] with the *M. tuberculosis* reference genome H37Rv (NC_000962.3). Quality control of the alignment was performed using QuallyMap v2.2.1 [6] for coverage statistics and Phred score to assess depth and coverage at all loci of interest associated with resistance. Only genomes> 75X deep and> 95% reference mapped to all UVP v2.7.0 loci of covered interest were considered for further analysis. Variant calling was accomplished with the use of GATK v4.2.0.0 [20] with filter adjustments to eliminate artificial false positive variants. Finally, NGSEP v4.0 was used to score both SNV and INDEL. [8] When analyzing the annotated VCF file, it was possible to identify variants with low, medium, and high confidence (LR +>10; p <0.05)” from **De novo assembly and annotation** section. The authors can write, “The variants obtained after variant calling were considered for the prediction of drug reistance” Line nos. 144-154; Page No. 7.

3. **Result**

**3.3 WGS prediction of drug resistance**

1. Asn927Asn is not non-synonymous mutation. Please check it, Line nos. 223; Page No. 11.
2. In Table 5. Variants related to drug resistance are listed. Out of 19, 42% variants are synonymous variants which should be excluded from the study as they will have no effect on the functional aspect of the gene.
4. **Discussion**
1. Should be read as, “these samples were” for “this sample was”, Line no. 257; Page No. 15.
2. “Other authors evaluated the variation that the protein present in these mutations could have and found that the effect of the mutation on it was neutral.” Rewrite this sentence. Line nos. 296-298; Page No. 16.
3. “The Asp103Asp mutation is a silent mutation in the rpoB gene reported by other authors in mono-resistant isolates”, Do this mutation affect the physiology of MTB? Line nos. 299-300; Page No. 17.
4. Should be read as, “in such a way this activity” for “in such a way that this activity” Line no. 323; Page No. 18.
5. Should be read as, “that allow to identify local” for “that allow identifying local” Line no. 333; Page No. 18.

**6. Conclusions**

1. Should be read as, “use of WGS for the characterization” for “use of the WGS the

characterization” Line no. 337; Page No. 18.

**Figure:**

**Figure. 1:** In the flow chart, in last box please check spelling of “analyses”.

**Tables**

**“Table 2. Clinical characteristics of pulmonary tuberculosis patients from Chinchiná, Colombia.”** is not present in the manuscript, Line no. 191; Page No. 9.
